# Whole-exome sequencing applications in prenatal diagnosis of fetal bowel dilatation

**DOI:** 10.1515/biol-2022-0598

**Published:** 2023-05-18

**Authors:** Xinyi Bian, Xiao Yang, Xinwei Shi, Wanjiang Zeng, Dongrui Deng, Suhua Chen, Fuyuan Qiao, Ling Feng, Yuanyuan Wu

**Affiliations:** Department of Obstetrics and Gynecology, Tongji Hospital, Tongji Medical College, Huazhong University of Science and Technology, No. 1095 Liberation Avenue, Wuhan 430030, Hubei, China

**Keywords:** whole-exome sequencing, fetal, bowel dilatation, pathogenic variants, prenatal diagnosis

## Abstract

This study introduced whole-exome sequencing (WES) in prenatal diagnosis of fetal bowel dilatation to improve the detection outcome when karyotype analysis and copy number variation sequencing (CNV-seq) were uninformative in detecting pathogenic variants. The work reviewed 28 cases diagnosed with fetal bowel dilatation and analyzed the results of karyotype analysis, CNV-seq, and WES. Among the 28 cases, the detection rate in cases with low risk of aneuploidy was 11.54% (3/26), which is lower than 100% (2/2) in cases with high risk of aneuploidy. Ten low-risk aneuploidy cases with isolated fetal bowel dilatation had normal genetic testing results, while the remaining 16 cases with other ultrasound abnormalities were detected for genetic variants at a rate of 18.75% (3/16). The detection rate of gene variation was 3.85% (1/26) by CNV-seq and 7.69% (2/26) by WES. This study suggested that WES could reveal more genetic risk in prenatal diagnosis of fetal bowel dilatation and has value in prenatal diagnosis to reduce birth defects.

## 1 Introduction

Fetal malformations and other structural abnormalities could be found through routine prenatal ultrasonographic examination in merely 3% of pregnancies, ranging from a single minor malformation, such as polydactyly, to severe multi-system abnormalities that are fatal [1,2]. Gastrointestinal malformation is one of the most common congenital malformations, accounting for about 7.35% in prenatal ultrasonographic examination and being manifested as fetal bowel dilatation [3]. However, due to the genetic heterogeneity and diverse clinical manifestations, the pathogenicity of fetal bowel dilatation is still unclear. It is important to identify the key genetic causes leading to fetal bowel dilatation, which could improve the accuracy in prenatal diagnosis.

Conventional karyotype analysis has been a first-line tool to investigate fetal abnormalities for more than 30 years. Subsequently, chromosomal microarray analysis (CMA) and copy number variation sequencing (CNV-seq) have been increasingly adopted in prenatal diagnosis to detect submicroscopic pathogenic copy number variations (pCNVs) in the past 10 years [1,4,5]. Currently, whole-exome sequencing (WES) is popularly used to investigate fetal anomaly showing good sequencing depth. Detection of karyotype analysis and CNV-seq has been often used to detect genetic causes of fetal bowel dilatation but provide little information. The additive application of WES in the prenatal diagnosis has been considered in modern genetic testing. This study combined WES analysis with karyotyping and CNV-seq to determine the genetic risks associated with fetal bowel dilatation.

## 2 Materials and methods

### 2.1 Participants and clinical data

This study reviewed 28 cases which were diagnosed with fetal bowel dilatation by prenatal ultrasonographic examination at Tongji Hospital affiliated to Tongji Medical College of Huazhong University of Science and Technology. Criterion for fetal bowel dilatation was fluid-filled intestinal loops at least 15 mm in length or 7 mm in diameter [6,7].

All participants received routine obstetric examinations, including ultrasound nuchal translucency screening at 11^+0^–13^+6^ weeks of gestation, non-invasive prenatal testing (NIPT) at 12–16 weeks of gestation, and ultrasonographic examination at 18–24 weeks of gestation. All cases were tested by karyotyping and CNV-seq. Cases confirmed with aneuploidy or pCNVs were not tested in the following WES analysis.


**Informed consent:** Informed consent has been obtained from all individuals included in this study.
**Ethical approval:** The research related to human use has been complied with all the relevant national regulations, institutional policies and in accordance with the tenets of the Helsinki Declaration, and has been approved by the authors’ institutional review board or equivalent committee (IRB ID: TJ-IRB20220104).

### 2.2 Genetic testing and laboratory evaluation

Amniocentesis was performed and 20 mL of amniotic fluid was sampled for karyotyping. The amniotic fluid was placed in an amniotic fluid culture bottle and grown in a carbon dioxide incubator. After 8 days of incubation, adherent cells were examined and treated with colchicine for 3–4 h. Next, cells were collected according to the standard laboratory procedures for G-band staining and karyotyping.

CNV-seq and WES were serviced by Berry Genomics. Trio-WES was performed to identify gene variations for the family trios. In brief, 1 μg of genomic DNA was extracted from 200 μL peripheral blood, using a Qiagen DNA Blood Midi/Mini kit (Qiagen GmbH, Hilden, Germany) following the manufacturer’s protocol. Fifty nanogram of DNA was fragmented to around 200 bp by enzyme treatment. The DNA fragments were end repaired by adding one A base at the 3′-end. Then, the DNA fragments were ligated with barcoded sequencing adaptors, and fragments at about 320 bp were collected by XP beads. After polymerase chain reaction (PCR) amplification, the DNA fragments were hybridized and captured by NanoWES according to the manufacturer’s protocol. The hybridized DNA products were collected by elution and subjected to PCR amplification and purification. Next, the libraries were quantified by quantitative real-time PCR (qPCR) and size distributions were determined using NanoWES (Berry Genomics, China). Novaseq6000 platform (Illumina, San Diego, USA), with 150 bp pair-end sequencing mode, was used for the family genomic DNA sequencing. Raw image files were processed using CASAVA v1.82 for base calling and generating raw data.

The sequencing reads were aligned to the human reference genome (hg19/GRCh37) using Burrows–Wheeler Aligner tool. PCR duplicates were removed by using Picard v1.57 (http://picard.sourceforge.net/). Verita Trekker® Variants Detection System by Berry Genomics and the third-party software GATK (https://software.broadinstitute.org/gatk/) were employed for variant calling. Variant annotation and interpretation were performed by ANNOVAR and the Enliven^®^ Variants Annotation Interpretation System referred to multiple databases including:

i) human population databases, such as gnomAD (http://gnomad.broadinstitute.org/), the 1000 Genome Project (http://browser.1000genomes.org), Berrybig data population database, dbSNP (http://www.ncbi.nlm.nih.gov/snp), etc.
ii) *in silico* prediction algorithms, such as SIFT (http://sift.jcvi.org), FATHMM (http://fathmm.biocompute.org.uk), Mutation Assessor (http://mutationassessor.org), CADD (http://cadd.gs.washington.edu), SPIDEX [8], etc.
iii) disease and phenotype databases, such as OMIM (http://www.omim.org), ClinVar (http://www.ncbi.nlm.nih.gov/clinvar), Human Gene Mutation Database (HGMD) (http://www.hgmd.org), HPO (https://hpo.jax.org/app/), etc.

The variants are classified into five categories, including pathogenic, likely pathogenic, uncertain significance, likely benign, and benign according to the American College of Medical Genetics and Genomics (ACMG) guidelines for interpretation of genetic variants [9]. Variants with minor allele frequencies <1% in exonic region or with splicing impact were assessed according to the identified ACMG category, inheritance model, available report on the pathogenicity, and recorded clinical synopsis [9–12].

## 3 Results

### 3.1 Study cohort

A total of 28 cases were included in this study, with 54% (*N* = 15) male fetuses and 45% (*N* = 13) female fetuses determined by karyotyping. The mean age of pregnant women in this cohort was 29 ± 4 years, and the mean gestational age was 27 weeks, with a range from 21 to 34 weeks. One case had a previous pregnancy with fetal bowel dilatation, while in the other 27 cases fetal bowel dilatation occurred for the first time.

Of the 28 cases, 57% (16/28) pregnancies resulted in live births, 39% (11/28) ended in pregnancy termination, and 4% (1/28) led to fetal demise. Karyotype analysis and CNV-seq were performed in all 28 cases. Twenty-five cases that showed negative results in karyotype analysis and CNV-seq were taken for WES analysis. There were three cases detected with pathogenic variants in karyotyping analysis and CNV-seq, and excluded from WES.

### 3.2 Detection of genetic variants in cases with different risks of aneuploidy

All the 28 pregnant women were examined with NIPT during pregnancy, of which 26 cases were found carrying low risk of aneuploidy and 2 cases had trisomy 21 at high risk. Two high-risk aneuploidy cases were detected with the presence of the “double-bubble” sign, indicating the duodenal atresia. There was one case out of the 26 low-risk aneuploidy cases detected with pCNVs. Twenty-five cases that showed negative results in karyotype analysis and CNV-seq were taken for WES, and genetic variants were detected in two cases. Differently, both karyotype analysis and CNV-seq suggested Down’s syndrome in two high-risk aneuploidy cases. Overall, the detection in cases with low risk of aneuploidy was 11.54% (3/26), while the detection in cases with high risk of aneuploidy was 100% (2/2) (Table 1).

**Table 1:** Number of cases detected at different risks of aneuploidy

Groups	Number of undetected cases, *n* (%)	Number of detected cases, *n* (%)	Total cases, *n* (%)
Low risk	23 (88.46)	3 (11.54)	26 (92.86)
High risk	0	2 (100.00)	2 (7.14)
Total	23 (82.14)	5 (17.86)	28 (100.00)

### 3.3 Detection of genetic variants in cases with other ultrasound abnormalities

Some cases with fetal bowel dilatation were also found to have other ultrasound abnormalities during the prenatal ultrasound examination, such as polyhydramnios, intestinal malrotation, and fetal echogenic bowel. There were 10 out of the 26 low-risk aneuploidy cases that showed isolated fetal bowel dilatation and had normal genetic testing results, while the remaining 16 cases with other ultrasound abnormalities were detected for genetic variants at a rate of 18.75% (3/16) (Table 2).

**Table 2:** Genetic variant detection of cases with other ultrasound abnormalities in the 26 low risk cases of aneuploidy

Groups	Number of undetected cases, *n* (%)	Number of detected cases, *n* (%)	Total cases, *n* (%)
Isolated fetal bowel dilatation	10 (100.00)	0	10 (38.48)
With other ultrasound abnormalities	13 (81.25)	3 (18.75)	16 (61.53)
Total	23 (88.46)	3 (11.54)	26 (100.00)

### 3.4 Genetic variant detection in cases at low risk of aneuploidy using WES

Among the 26 cases with low risk of aneuploidy, the application of karyotype analysis, CNV-seq, and WES were found in three cases carrying genetic variants. The karyotype analysis showed normal results. The CNV-seq detected microduplication of chromosome 15q26.1-q26.3 in one case, covering the entire region of 15q26 overgrowth syndrome. The phenotype of this case was typical. This case finally ended in pregnancy termination.

WES revealed genetic variants in two cases with fetal bowel dilatation. One case that resulted in fetal demise carried a heterozygous variant of glial cell-derived neurotrophic factor (*GDNF*) gene c.329G > A, inherited from his father. This mutation has not been reported previously and it can cause amino acid changes negatively affecting the bio-function of the protein. It is known that *GDNF* gene mutation can cause an autosomal dominant (AD) Hirschsprung’s disease 3. This disease is characterized by low penetrance [13]. The mother of the case reported that her last pregnancy happened 2 years ago and revealed bowel dilatation in ultrasound examination. The newborn showed non-defecation with vomiting yellow-green secretions after birth, and eventually died 1 month later. No genetic examination was conducted in her last pregnancy. According to the medical history, it was speculated that this mutation led to fetal bowel dilatation in this case.

Another case that had pregnancy termination was identified with a compound heterozygous variant in the solute carrier family 26 member 3 gene (*SLC26A3*), suggesting a c.1427del variant from the father and a c.269_270dup variant from the mother. The gene mutation can cause autosomal recessive (AR) inherited congenital chloride diarrhea 1. The c.1427del variant was not seen in current genomic databases. The mutation could swift gene open reading frame leading to damage protein function, indicating as strong pathogenicity PVS1 and moderate pathogenicity PM2. The mutation was listed in the Shenzhou Genome database, the human Exon Database (ExAC), the thousand genomes of the reference population (1,000 G), and the Population Genome Mutation Frequency Database (gnomAD). The moderate pathogenicity PM3 is suggested to constitute a complex heterozygote with the mutation site of c.269_270dup. The c.269_270dup variant was also determined as pathogenic (PVS1 + PM2 + PM3) and previously reported as likely pathogenic in the ClinVar database and disease-causing mutation in HGMD [14]. Both variants could lead to protein dysfunction and are considered pathogenic according to ACMG guidelines (Table 3) [9].

**Table 3:** Genetic variants identified by WES in two cases with fetal bowel dilatation

Case no.	Gene	RefSeq identifier	DNA alteration	Protein alteration	Origin	ACMG classification	Inheritance	Diagnosis
1	*GDNF*	NM_001190468.1	chr5: 37816111, c.329G > A	Arg110Gln	Paternal inherited	Uncertain significance	AD	Hirschsprung’s disease 3
2	*SLC26A3*	NM_000111.3	chr7:1077 78262-10 7778262, c.1427del; chr7:1077 93742-10 7793742, c.269_270dup	Phe476Ser; Gly91Lys	Compound heterozygous	Pathogenic	AR	Congenital chloride diarrhea 1

Overall, the detection rate of genetic variants was 3.85% (1/26) by CNV-seq and 7.69% (2/26) by WES (Table 4).

**Table 4:** Detection by different methods presented in the 26 low risk cases of aneuploidy

Prenatal diagnosis method	Number of detected cases	Detection rate (%)
Karyotype analysis	0	0
CNV-seq	1	3.85
WES	2	7.69
Total	3	11.54

### 3.5 Detection rate of different prenatal diagnosis methods in cases with other ultrasound abnormalities

Among the 16 cases with other ultrasound abnormalities, 5 cases had polyhydramnios, and two of them were detected with pathogenic variants by WES, at a detection rate of 40% (2/5). In other 11 cases, only 1 case was detected with pCNVs by CNV-seq, at a detection rate of 9.09% (1/11) (Table 5).

**Table 5:** Detection rate of different prenatal diagnosis methods in the 16 cases with other ultrasound abnormalities and at low-risk aneuploidy

Prenatal diagnosis methods	Polyhydramnios (*n* = 5)		Other ultrasound abnormalities (*n* = 11)	
	Number of detected cases	Detection rate (%)	Number of detected cases	Detection rate (%)
Karyotype analysis	0	0	0	0
CNV-seq	0	0	1	9.09
WES	2	40.00	0	0
Total	2	40.00	1	9.09

## 4 Discussion

Genetic diseases are complex in many forms, including chromosomal abnormalities, monogenic mutations, polygenic mutations, and mitochondrial diseases [15]. These diseases may cause intellectual disabilities, congenital anomalies, and even death. In recent years, the development of prenatal diagnosis and ultrasound technology improved the detection rate of fetal congenital malformations, and some genetic diseases can be confirmed in obstetric examinations. Therefore, timely and accurate prenatal diagnosis is essential for preventing birth defects.

Current evidence showed that chromosomal abnormalities were associated with fetal bowel dilatation. Chromosomal abnormalities have been reported in approximately one-third of duodenal dilatation cases, mainly trisomy 21 [16,17]. In this study, cases at high-risk aneuploidy by NIPT were identified with pathogenic variants at a detection rate of 100%, while cases in low-risk aneuploidy had lower detection rate of 11.54%. This suggests that chromosomal abnormalities are highly suspected in cases with fetal bowel dilatation at high-risk aneuploidy, and gene examination should be recommended in prenatal diagnosis. In cases with fetal bowel dilatation at low-risk aneuploidy, further analysis is needed to determine whether gene examination is necessary. In addition, two high-risk aneuploidy cases were diagnosed as trisomy 21, and both of them were detected with the presence of the “double-bubble” sign, indicating the duodenal atresia, which was consistent with the previous findings.

Fetal bowel dilatation is mostly caused by obstructive bowel diseases, such as intestinal atresia and stenosis, congenital intestinal malrotation, meconium ileus, and cystic fibrosis [18–20]. Previous studies have found that intestinal malformation occurred less frequently in isolated bowel dilatation than in bowel dilatation in combination with other abnormal ultrasound features [19]. For the detection rate of genetic variants, the differences between the two groups are rarely evaluated in the existing studies. In this study, the observation is that fetuses with other ultrasound abnormalities are more likely to have pathogenic variants. The detection rate increased from 11.54 to 18.75%. It indicated that gene examination may be more necessary in low-risk aneuploidy cases combined with other ultrasound abnormalities. The isolated fetal bowel dilatation cases showed normal genetic testing results, suggesting that it may be related to transient bowel dilatation or structural variation rather than a genetic disorder.

Gene examination mainly uses karyotyping for detecting chromosomal abnormalities, and CMA or CNV-seq for identifying microdeletions and microduplications. Recently, exome sequencing appears to be a promising technique for its increased diagnostic accuracy when karyotyping and microarrays show normal results. Most identified genes implicated in Mendelian disease often are present in exons [21,22]. Petrovski et al. found that in pregnancies with fetal structural anomalies, WES could identify genetic variants in 10% cases when karyotyping and CMA showed normal results [23]. The application of WES in this investigation also improved the diagnostic yield for the genetic variants. Two cases that showed negative results in karyotype analysis and CNV-seq were identified with genetic variants by WES, showing an incremental yield of 7.69%. These two pathogenic variants cause Hirschsprung’s disease 3 and congenital chloride diarrhea 1. Therefore, WES may have good application value in the prenatal diagnosis of fetal bowel dilatation, especially for rare genetic disorders, which can improve the diagnosing accuracy. In addition, our study also found that cases with fetal bowel dilatation and polyhydramnios carried pathogenic variants detected by WES at higher likelihood (40%). It indicated that WES combined with other ultrasound abnormalities could be a more effective and accurate approach for prenatal diagnosis, especially combined with polyhydramnios, and consequently reducing the occurrence of birth defects.

The findings suggested that in cases with fetal bowel dilatation at high-risk aneuploidy, gene examination should be recommended in prenatal diagnosis. Meanwhile, in cases at low-risk aneuploidy, WES could reveal more genetic risks in prenatal diagnosis of fetal bowel dilatation and has value in prenatal diagnosis to reduce birth defects, particularly in cases combined with other ultrasound abnormalities. In prenatal genetic counseling, pregnant women should be informed of the possible risk of genetic disorders, and it is up to them to decide whether to receive WES analysis.

## References

[1] Lord J, McMullan DJ, Eberhardt RY, Rinck G, Hamilton SJ, Quinlan-Jones E, et al. Prenatal exome sequencing analysis in fetal structural anomalies detected by ultrasonography (PAGE): a cohort study. Lancet. 2019;393(10173):747–57. 10.1016/S0140-6736(18)31940-8 PMC6386638 30712880

[2] Persson M, Cnattingius S, Villamor E, Soderling J, Pasternak B, Stephansson O, et al. Risk of major congenital malformations in relation to maternal overweight and obesity severity: cohort study of 1.2 million singletons. BMJ. 2017;357:j2563. 10.1136/bmj.j2563 PMC5470075 28615173

[3] Carrera JM, Torrents M, Mortera C, Cusi V, Munoz A. Routine prenatal ultrasound screening for fetal abnormalities: 22 years’ experience. Ultrasound Obstet Gynecol. 1995;5(3):174–9. 10.1046/j.1469-0705.1995.05030174.x 7788491

[4] Robson SC, Chitty LS, Morris S, Verhoef T, Ambler G, Wellesley DG, et al. Evaluation of array comparative genomic hybridisation in prenatal diagnosis of fetal anomalies: A multicentre cohort study with cost analysis and assessment of patient, health professional and commissioner preferences for array comparative genomic hybridisation. Efficacy Mech Eval. Southampton (UK). 2017;4(1):1–104. 28182369

[5] Wapner RJ, Martin CL, Levy B, Ballif BC, Eng CM, Zachary JM, et al. Chromosomal microarray versus karyotyping for prenatal diagnosis. N Engl J Med. 2012;367(23):2175–84. 10.1056/NEJMoa1203382 PMC3549418 23215555

[6] Andrade WS, Brizot ML, Francisco RPV, Tannuri AC, Syngelaki A, Akolekar R, et al. Fetal intra-abdominal bowel dilation in prediction of complex gastroschisis. Ultrasound Obstet Gynecol. 2019;54(3):376–80. 10.1002/uog.20367 31264279

[7] Nyberg DA, Mack LA, Patten RM, Cyr DR. Fetal bowel. Normal sonographic findings. J Ultrasound Med. 1987;6(1):3–6. 10.7863/jum.1987.6.1.3 3546721

[8] Xiong HY, Alipanahi B, Lee LJ, Bretschneider H, Merico D, Yuen RK, et al. RNA splicing. The human splicing code reveals new insights into the genetic determinants of disease. Science. 2015;347(6218):1254806. 10.1126/science.1254806 PMC4362528 25525159

[9] Richards S, Aziz N, Bale S, Bick D, Das S, Gastier-Foster J, et al. Standards and guidelines for the interpretation of sequence variants: a joint consensus recommendation of the American College of Medical Genetics and Genomics and the Association for Molecular Pathology. Genet Med. 2015;17(5):405–24. 10.1038/gim.2015.30 PMC4544753 25741868

[10] Abou Tayoun AN, Pesaran T, DiStefano MT, Oza A, Rehm HL, Biesecker LG, et al. Recommendations for interpreting the loss of function PVS1 ACMG/AMP variant criterion. Hum Mutat. 2018;39(11):1517–24. 10.1002/humu.23626 PMC6185798 30192042

[11] Biesecker LG, Harrison SM. ClinGen Sequence Variant Interpretation Working G. The ACMG/AMP reputable source criteria for the interpretation of sequence variants. Genet Med. 2018;20(12):1687–8. 10.1038/gim.2018.42 PMC6709533 29543229

[12] Ghosh R, Harrison SM, Rehm HL, Plon SE, Biesecker LG. ClinGen Sequence Variant Interpretation Working G. Updated recommendation for the benign stand-alone ACMG/AMP criterion. Hum Mutat. 2018;39(11):1525–30. 10.1002/humu.23642 PMC6188666 30311383

[13] Amiel J, Sproat-Emison E, Garcia-Barcelo M, Lantieri F, Burzynski G, Borrego S, et al. Hirschsprung disease, associated syndromes and genetics: a review. J Med Genet. 2008;45(1):1–14. 10.1136/jmg.2007.053959 17965226

[14] Wedenoja S, Pekansaari E, Hoglund P, Makela S, Holmberg C, Kere J. Update on SLC26A3 mutations in congenital chloride diarrhea. Hum Mutat. 2011;32(7):715–22. 10.1002/humu.21498 21394828

[15] Roth TL, Marson A. Genetic disease and therapy. Annu Rev Pathol. 2021;16:145–66. 10.1146/annurev-pathmechdis-012419-032626 PMC9704033 33497260

[16] Bethell GS, Long AM, Knight M, Hall NJ, Baps C. The impact of trisomy 21 on epidemiology, management, and outcomes of congenital duodenal obstruction: a population-based study. Pediatr Surg Int. 2020;36(4):477–83. 10.1007/s00383-020-04628-w PMC7069923 32114651

[17] Galani A, Zikopoulos A, Papandreou L, Mastora E, Zikopoulos K, Makrydimas G. Prenatal diagnosis of fetal jejunal atresia: a case report. Cureus. 2021;13(10):e18947. 10.7759/cureus.18947 PMC8605830 34815896

[18] Dewberry LC, Hilton SA, Zaretsky MV, Behrendt N, Galan HL, Marwan AI, et al. Examination of prenatal sonographic findings: intra-abdominal bowel dilation predicts poor gastroschisis outcomes. Fetal Diagn Ther. 2020;47(3):245–50. 10.1159/000501592 31454815

[19] Xu S, Zhong W, Shen Z, Dai C, Xia H, Guan Q, et al. Analysis of the clinical outcomes of fetal bowel dilatation combined with other abnormal ultrasonographic features. J Matern Fetal Neonatal Med. 2019;32(6):992–6. 10.1080/14767058.2017.1397123 29113511

[20] Katz G, Pode-Shakked B, Berkenstadt M, Bilik R, Polak Charcon S, Barshack I, et al. Nonobstructive diffuse dilated bowel loops: prenatal diagnosis, fetal characteristics and neonatal outcomes. J Ultrasound Med. 2017;36(1):149–54. 10.7863/ultra.16.01097 27933652

[21] Jelin AC, Vora N. Whole exome sequencing: applications in prenatal genetics. Obstet Gynecol Clin North Am. 2018;45(1):69–81. 10.1016/j.ogc.2017.10.003 PMC5813701 29428287

[22] Majewski J, Schwartzentruber J, Lalonde E, Montpetit A, Jabado N. What can exome sequencing do for you? J Med Genet. 2011;48(9):580–9. 10.1136/jmedgenet-2011-100223 21730106

[23] Petrovski S, Aggarwal V, Giordano JL, Stosic M, Wou K, Bier L, et al. Whole-exome sequencing in the evaluation of fetal structural anomalies: a prospective cohort study. Lancet. 2019;393(10173):758–67. 10.1016/S0140-6736(18)32042-7 30712878

